# Metabolic pathway alignment between species using a comprehensive and flexible similarity measure

**DOI:** 10.1186/1752-0509-2-111

**Published:** 2008-12-24

**Authors:** Yunlei Li, Dick de Ridder, Marco JL de Groot, Marcel JT Reinders

**Affiliations:** 1 Information and Communication Theory Group, Faculty of Electrical Engineering, Mathematics and Computer Science, Delft University of Technology, Mekelweg 4, 2628 CD Delft, the Netherlands; 2 Netherlands Bioinformatics Centre, 260 NBIC, P.O. Box 9101, 6500 HB Nijmegen, the Netherlands; 3 Kluyver Centre for Genomics of Industrial Fermentation, Julianalaan 67, 2628 BC Delft, the Netherlands; 4 Bioprocess Technology Group, Department of Biotechnology, Faculty of Applied Sciences, Delft University of Technology, Julianalaan 67, 2628 BC Delft, the Netherlands

## Abstract

**Background:**

Comparative analysis of metabolic networks in multiple species yields important information on their evolution, and has great practical value in metabolic engineering, human disease analysis, drug design etc. In this work, we aim to systematically search for conserved pathways in two species, quantify their similarities, and focus on the variations between them.

**Results:**

We present an efficient framework, Metabolic Pathway Alignment and Scoring (M-PAS), for identifying and ranking conserved metabolic pathways. M-PAS aligns all reactions in entire metabolic networks of two species and assembles them into pathways, taking mismatches, gaps and crossovers into account. It uses a comprehensive scoring function, which quantifies pathway similarity such that we can focus on different pathways given different biological motivations. Using M-PAS, we detected 1198 length-four pathways fully conserved between *Saccharomyces cerevisiae *and *Escherichia coli*, and also revealed 1399 cases of a species using a unique route in otherwise highly conserved pathways.

**Conclusion:**

Our method efficiently automates the process of exploring reaction arrangement possibilities, both between species and within species, to find conserved pathways. We not only reconstruct conventional pathways such as those found in KEGG, but also discover new pathway possibilities. Our results can help to generate hypotheses on missing reactions and manifest differences in highly conserved pathways, which is useful for biology and life science applications.

## Background

Comparative analysis of metabolic networks in different species yields information important for both biology (understanding evolution/speciation, annotating new genomes etc.) and life science applications (e.g. in biotechnology, pharmacology). Therefore, it has been an active research field for the last decade. For example, Dandekar *et al*. [1] combined biochemical data analysis, elementary flux mode analysis and comparative genome analysis to compare glycolytic pathways in 17 species. Jeong *et al*. [2] and Ravasz *et al*. [3] studied the global topological properties of the metabolic networks in 43 species. In addition, Küffner *et al*. [4] used Petri nets to compare database contents and define differential metabolic displays (DMDs), which allow to compare metabolic networks by identifying intersection and difference sets of reactions. As one of the applications, Heymans *et al*. [5] derived phylogenetic trees based on metabolic pathway comparison. Guimerà *et al*. [6] analyzed the modularity of the metabolic networks of 18 organisms, and classified metabolites and enzymes based on their roles in connecting different functional modules. Díaz-Mejía *et al*. [7] investigated the relation of network modularity and distance between reactions with the retention of gene duplicates in various species and databases. More generally, a review on biological network comparison problems, techniques and applications is given by Sharan *et al*. [8].

In studies up till now, however, only little work focused explicitly on the variations between species in conserved pathways, and to our knowledge no alignment of entire networks, exploiting all reaction arrangement possibilities, has been carried out yet. Moreover, the similarity measures used to align metabolic pathways is often not comprehensive, as compounds or network structure are neglected. For example, Tohsato *et al*. [9] align pathways based on enzyme EC number similarity only, discarding information on the compounds involved. Yang *et al*. [10] perform path matching and graph matching to query certain metabolic pathways or subgraphs in a predefined graph, but also use a similarity measure based on EC numbers only. Although Forst *et al*. [11] define the distance between pathways as a combination of distances between compounds and distances between enzymes, they only consider sequence similarity, and the compounds are limited to amino acids. In [12], sets of reactions in multiple pathways are compared, omitting the connectivity between the reactions. Finally, the pathway similarity score in [5,13,14] combines EC number similarity and network topology, but does not include compounds, and alignments are between predefined sub-networks only. Therefore, the comparison is limited to conventional pathways, and different parts of the cellular metabolism are not associated with each other.

**Figure 1:**
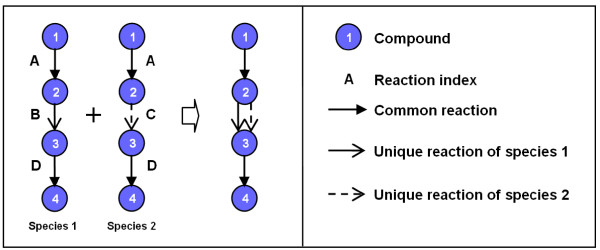
**Illustration of our searching target**. The pathways in two species share common reactions (A and D), but also have variations (B and C).

In this work, we align entire metabolic networks of two species and quantify their similarities comprehensively, to identify highly conserved pathways. We particularly focus on the variations in these pathways, as illustrated in Figure 1. In this paper, a pathway is defined as a series of chemical reactions of metabolism within a cell. Therefore they are not necessarily routes through the network from uptake to secretion, as represented by many conventional pathway representations.

A naive approach to find conservation and variations between metabolic networks would be to search for common reactions and reaction pairs, using different cofactors or enzymes in the two species. Besides being inefficient, this approach isolates reactions from their upstream and downstream processes. Instead, we search for conserved *pathways*, rather than single *reactions*. In this way, we place the reactions in their metabolic functional context, which helps to 1) filter out isolated reactions not involved in pathways, 2) provide more evidence to claim part of a pathway is conserved, given that neighboring reactions are conserved, 3) interpret the resulting pathways.

**Figure 2:**
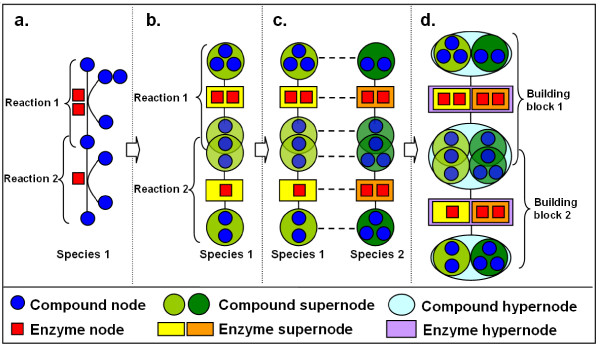
**Overview of the method**. First, compound nodes and enzyme nodes (**a**) are generalized into compound supernodes and enzyme supernodes (**b**). Two reactions of species 1 are aligned with two reactions of species 2 (**c**), by pairing the supernodes into compound hypernodes and enzyme hypernodes (**d**). Each pair of aligned reaction forms a building block, from which an aligned pathway can be assembled. The reaction directions are omitted in the figure for simplicity.

Our method is designed to conduct this process efficiently and comprehensively. More specifically, our pairwise pathway alignment is based on a mechanism we proposed earlier [15], which is inspired by the alignment concept of [16]. It first aligns two to four similar reactions in two species into building blocks, and then assembles these into pathways of a desired length (Figure 2). In each building block, a specific substrate is transformed into a specific product via similar but not necessarily identical reactions in two species. That is, they may have different co-substrates or co-products, be catalyzed by different enzymes, need different numbers of reactions to complete the transformation, or reactions may occur in a different order. In other words, our method enables to explore topological arrangement possibilities of reactions both between species (by building block assembly) and within species (by pathway assembly).

Further, we rank the aligned pathways according to their similarities (i.e. level of conservation), which prioritizes them for further investigation. To this end, a novel scoring function is proposed, which forms the core contribution of this paper. It compares all components of two pathways by measuring similarities between substrate sets, product sets, enzyme functions, enzyme sequences, and alignment topology. The resulting individual similarity measures are then integrated into a single score. This scoring function has a generic form and is flexible enough to address various biological questions, by selecting different parameter settings.

## Results

### Algorithm

We align the pathways from two species in a strict way, in order to investigate highly conserved metabolic pathways, i.e. pathways with very similar structure and limited variation between species. More specifically, two metabolic pathways can be aligned into a conserved pathway only if their individual reactions transform common substrates into common products in each step. We call such a pair of matching reactions a building block (BB). Next, these building blocks are assembled into pathways of a specified length, taking reaction directions into account. Finally, we compute the similarity score for each aligned pathway, and obtain interesting pathways as those pathways that have high similarity scores.

#### Reaction representation

In M-PAS, reactions are represented at three levels of generalization: nodes, supernodes and hypernodes, respectively (see Figure 2). The low-level representation gives the finest details of reactions, in which each compound and each enzyme constitutes a node (Figure 2a). The medium-level representation generalizes reactions, so that all substrates and products of a reaction compose two compound supernodes, and all enzymes in that reaction form an enzyme supernode (Figure 2b). Such a generalized representation is useful due to the multiple-to-multiple property of metabolic reactions, i.e. multiple substrates can be catalyzed by multiple enzymes into multiple products [8,17]. Finally, at the high-level representation, the corresponding compound supernodes and enzyme supernodes from two aligned reactions are combined into compound hypernodes and enzyme hypernodes, respectively (Figure 2c–d).

These different levels of representation enable the comparison of reactions in a detailed yet flexible manner. Thus, a particular compound node can be part of various compound supernodes given different co-factors in different reactions, and further can be part of various compound hypernodes due to different alignments with other compound supernodes. The same holds for enzyme nodes. This flexible representation not only reflects the versatility of the metabolic network conveniently, but is also necessary in order to express and quantify the similarity of reactions, which will be explained in the section *Scoring function*.

#### Reaction alignment

**Figure 3:**
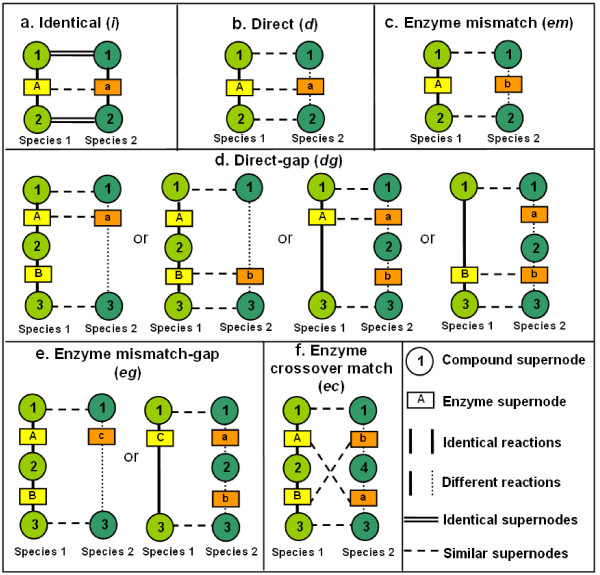
**Illustration of the six types of building blocks**. The reaction directions are omitted in the figure for simplicity. The color legends are the same as in Figure 2. Two compound supernodes are considered similar if they share at least one common compound node. Two enzyme supernodes are considered similar if there exists a pair of enzymes which share the same first two digits in their EC numbers.

The reaction alignment part is proposed in our previous work [15] and is briefly explained here for comprehensibility and completeness of our methodology. Two reactions can be aligned to form a building block when they have at least one common substrate node and one common product node (Figure 2d). To allow for some variation, we introduce six types of building blocks (see Figure 3). If the same reaction is present in both species, the resulting building block is called "identical" (*i*). If the two reactions are different, but the first two digits of the EC numbers of their enzymes are the same, they form a "direct" building block (*d*).

We allow for up to one mismatch or one gap in a building block, in order to incorporate alternative pathways, evolutionary diversity and annotation errors. That is, in an "enzyme mismatch" building block (*em*), the first two digits of the EC numbers of their enzymes are not the same. Gaps occur when a single reaction and a series of reactions connected in tandem share common substrates and products, indicating that the number of reactions to transform the specific substrates into the specific products differs between species. The building blocks containing one gap are "direct-gap" (*dg*) and "enzyme mismatch-gap" (*eg*). Finally, we include "enzyme crossover match" building blocks (*ec*) to accommodate possible variations in the order of the catalysis. That is, apart from sharing common substrates and end products in two reactions in each species, the first two EC number digits of the first and second reaction in one species are the same as those of the second and first reaction in the other species, respectively.

To enhance the informativeness of these resulting pathways, we add a constraint to avoid redundant building blocks. That is, a non-identical building block can be constructed only if it contains at least one unique reaction in one of the species, which is absent in the other species. This is because if two reactions converting the same substrate into the same product (e.g. A and B) are present in both species, two "identical" building blocks *A*_1_-*A*_2 _and *B*_1_-*B*_2 _are constructed already. Therefore, any other combinations of these reactions (i.e. *A*_1_-*B*_2 _and *B*_1_-*A*_2_) are just worse matches.

#### Scoring function

We set out by specifying a number of criteria for the design of the scoring function. First, similarities of all reaction components should be considered: substrate sets, product sets, enzyme functions and enzyme sequences, respectively. Second, the scoring function should be flexible and adaptable according to the user's biological interests. For example, the user might want to find pathways containing a particular structure (e.g. with a gap); or focus on enzymes only, but not on compounds; or seek to find a completely alternative pathway in which the enzymes are very dissimilar between two species. Third, since we aim to investigate many aspects of an aligned pathway and obtain multiple similarity scores, a reasonable way of integrating these is required. Finally, we should consider specificity in computing similarities, since both distributions of compound connectivity and enzyme EC number hierarchy show large variation [9,18], i.e. some compounds and EC subclasses appear more often than the others in the background.

#### 1) Total score

According to the criteria above, we first compute similarity scores independently for all compound hypernodes and enzyme hypernodes in an aligned pathway, taking all aspects into account. These are then converted into *z*-scores before integration to account for their diverse distributions.

Let *Z*(*x*) denote the *z*-score of *x*. Then *Z*(*P*) is the total *z*-score for an aligned pathway *P*, a weighted sum of the scores of *N *building blocks *B *in *P*:

(1) Z(P)=12N∑∀B∈P[Z0(B)+Z(B)]=12N∑∀B∈P[Z0(B)+1ωc2+ωe2(ωcZ(CB)+ωeZ(EB))]

*Z*(*B*) is the *z*-score for a building block *B*. Let *c *and *e *denote a compound hypernode and an enzyme hypernode respectively, and denote the set of all *c*'s and *e*'s in a building block *B *by *C*_*B *_and *E*_*B*_, respectively. Users can define a preferred building block structure by assigning different biases (*Z*_0_(*B*)) to different building block types. For example, if building blocks with gaps are preferred in a query, then these types of building block can be assigned a large positive bias. Weights *ω*_*c*_, *ω*_*e *_∈ [0, 1] can be used to assign different relative importance to compound similarity and enzyme similarity (resembling the *α *parameter in [12]).

Note that the *z*-scores are hierarchically combined using Liptak-Stouffer's method [19,20]. In the following we explain how to compute *Z*(*C*_*B*_) and *Z*(*E*_*B*_) in detail.

#### 2) Compound similarity

*Z*(*C*_*B*_) is composed of compound similarities *Z*(*c*) of the two compound hypernodes in the building block (i.e. the substrate hypernode and product hypernode). We express *Z*(*c*) in two terms:

(2) $$Z(C_{B})=\frac{1}{\sqrt{2}}\sum_{\forall c\in C_{B}}Z(c)=\frac{1}{\sqrt{2}}\sum_{\forall c\in C_{B}}\frac{1}{\sqrt{2}}[Z_{A}(c)+Z_{S}(c)]$$

The *agreement Z*_*A*_(*c*) is the extent of the overlap in number of compounds between the two aligned compound supernodes. This is computed as the probability of observing the amount of overlap between the two compound supernodes by chance, according to a hypergeometric distribution [21]:

(3) $$P_{A}(c)=\frac{\left(\begin{array}{c} \left|c_{1}\right|\\ \left|c_{1}\cap c_{2}\right| \end{array}\right)\left(\begin{array}{c} \left|c_{1}\cup c_{2}\right|-\left|c_{1}\right|\\ \left|c_{2}\right|-\left|c_{1}\cap c_{2}\right| \end{array}\right)}{\left(\begin{array}{c} \left|c_{1}\cup c_{2}\right|\\ \left|c_{2}\right| \end{array}\right)}=\frac{\left(\begin{array}{c} \left|c_{1}\right|\\ \left|c_{1}\cap c_{2}\right| \end{array}\right)}{\left(\begin{array}{c} \left|c_{1}\cup c_{2}\right|\\ \left|c_{2}\right| \end{array}\right)}$$

where *c*_1 _and *c*_2 _denote the compound supernodes that form *c*, and |*x*| denotes the number of compound nodes in *x*.

Next, this probability is transformed to a *z*-score:

(4) $$Z_{A}(c)=\frac{P_{A}(c)-\mu_{AC}}{\sigma_{AC}}$$

where *μ*_*AC *_and *σ*_*AC *_are the mean and standard-deviation of *P*_*A*_(*c*) over all possible compound supernode pairs, which represent the expected amount of overlap when the pairing would be random.

The other term is *Z*_*S*_(*c*), the *specificity *of the overlap when compared to all possible supernode pairs. That is, if two compound supernodes have overlapping compounds, we take into account the frequency of obtaining this particular overlap at random. We consider two sets of substances to be more similar if the overlapping part is more specific, i.e. not observed frequently by chance. Moreover, considering specificity of compounds may result in more biologically meaningful pathways, since metabolic pathways seem to represent paths through the least "promiscuous" compounds [18].

Suppose there are in total *m *compound supernodes in species 1 and *n *in species 2. Then we have:

(5) $$P_{S}(c)=1-\frac{\#\text{observed }(c_{1}\cap c_{2})\text{ in the intersection}}{mn}$$

(6) $$Z_{S}(c)=\frac{P_{S}(c)-\mu_{SC}}{\sigma_{SC}}$$

where *μ*_*SC *_and *σ*_*SC *_are the mean and standard-deviation of *P*_*S*_(*c*) computed over all *mn *compound supernode pairs. The numerator in (5) is the number of times the specific overlap in compound node in *c*, i.e. (*c*_1 _∩ *c*_2_), is observed in the intersections of all possible compound supernode pairs.

#### 3) Enzyme similarity

The enzyme hypernode similarity score, *Z*(*E*_*B*_), is defined by a functional similarity score *Z*_*F*_(*e*) and a sequence similarity score *Z*_*Q*_(*e*). In addition, users can specify weights *ω*_*f*_, *ω*_*q *_∈ [-1, 1] for the functional and sequence similarity scores to indicate their relative importance. Setting these weights to negative values actually enables us to search for dissimilar enzymes, which associates reactions with different mechanisms and provides more possibilities to annotate new species. For generality, suppose there are *k *enzyme hypernodes in building block *B *(*k *= 2 for "enzyme crossover match" building blocks, *k *= 1 for others). The enzyme similarity is then given by:

(7) Z(EB)=1k∑∀e∈EBZ(e)=1k∑∀e∈EB1ωf2+ωq2[ωfZF(e)+ωqZQ(e)]

*Z*_*F*_(*e*) is computed similar to (2)–(6), containing agreement and specificity of the EC number overlap:

(8) $$Z_{F}(e)=\frac{1}{\sqrt{2}}[Z_{A}(e)+Z_{S}(e)]$$

The enzyme functional agreement score *Z*_*A*_(*e*) is derived from *P*_*A*_(*e*), the probability of obtaining by chance the number of common subclasses between the EC numbers of *e*_1 _and *e*_2_, the two enzyme supernodes that form hypernode *e*. Let T denote the set of all subclasses, and ℳ be the overlapping subclasses. For instance, for *e*_1 _= 1.2.3.4 and *e*_2 _= 1.2.4.4, T = {1, 1.2, 1.2.3, 1.2.4, 1.2.3.4, 1.2.4.4}, and ℳ = {1, 1.2}. These sets are then used to assess the extent of overlap between two EC numbers, analogous to (3):

(9) $$P_{A}(e)=\frac{\left(\begin{array}{c} 4\\ \left|ℳ\right| \end{array}\right)\left(\begin{array}{c} \left|T\right|-4\\ 4-\left|ℳ\right| \end{array}\right)}{\left(\begin{array}{c} \left|T\right|\\ 4 \end{array}\right)}=\frac{\left(\begin{array}{c} 4\\ \left|ℳ\right| \end{array}\right)}{\left(\begin{array}{c} \left|T\right|\\ 4 \end{array}\right)}$$

(10) $$Z_{A}(e)=\frac{P_{A}(e)-\mu_{AE}}{\sigma_{AE}}$$

where *μ*_*AE *_and *σ*_*AE *_are computed from *P*_*A*_(*e*) over all possible enzyme supernode pairs.

To address the specificity of the observed ℳ, we also count the number of times the common EC number subclasses of two enzyme supernodes contains this ℳ, and compute *P*_*S*_(*e*), *μ*_*SE*_, *σ*_*SE *_and *Z*_*S*_(*e*), analogous to (5)–(6):

(11) $$P_{S}(e)=1-\frac{\#\text{observed }ℳ\text{ in the overlapping subclasses}}{uv}$$

(12) $$Z_{S}(e)=\frac{P_{S}(e)-\mu_{SE}}{\sigma_{SE}}$$

with *u *and *v *the total numbers of enzyme supernodes in the two species.

Finally, the sequence similarity score *Z*_*Q*_(*e*) is derived from the BLAST *E *-value *L*(*e*):

(13) $$\begin{array}{cc} Q(e)=-log_{10}L(e), & Z_{Q}(e)=\frac{Q(e)-\mu_{q}}{\sigma_{q}} \end{array}$$

where *μ*_*q *_and *σ*_*q *_are the mean and standard-deviation of *Q*(*e*) over all possible enzyme supernode pairs.

Note that there might exist multiple EC numbers and multiple sequences in each enzyme supernode, as illustrated in Figure 2. So we first compute all *Z*(*e*) given all possible combinations of EC numbers and corresponding sequences in enzyme hypernode e. Since we aim to find the conserved part between pathways, the highest *Z*(*e*) is taken to be the enzyme similarity score for this pair of supernodes, indicating the similarity of the most conserved part between them.

Moreover, when gaps are present, we align two enzyme supernodes in one species with one enzyme supernode in another species separately, obtaining two *Z*(*e*). Again, the higher one is selected for this building block to represent the similarity of the most conserved part.

#### Pathway construction

Reaction definitions were obtained from Release 42.0 of the KEGG LIGAND composite database [22], updated on May 14, 2007. The species-specific reactions and enzyme lists were retrieved from KEGG/XML and KEGG/PATHWAY. Protein sequences were downloaded from UniProtKB/Swiss-Prot [23]. Discrepancies and missing information (e.g. gene names and EC numbers) were resolved manually. Twenty-six currency metabolites (see Appendix) are excluded from consideration during pathway construction to avoid finding large numbers of pathway shortcuts [3,24,25]. Note that the reactions containing these metabolites are still included in the algorithm. Currency metabolites are only excluded in aligning reactions into building blocks and assembling pathways, i.e. we do not match or connect two reactions if they only share the same currency metabolites.

Based on 881 enzymatic reactions in *S. cerevisiae *(with 1762 compound supernodes and 881 enzyme supernodes) and 1106 enzymatic reactions in *E. coli *(with 2212 compound supernodes and 1106 enzyme supernodes), 640 building blocks are constructed. These are further concatenated into pathways using a backtracking search, starting from a certain substrate. Each pathway contains four different building blocks, and is constrained so that one reaction cannot appear more than once in one species, and one compound (excluding the currency metabolites) cannot be traversed more than once in one species, e.g. a compound can not be both the substrate and product of a reaction, or be the products of two reactions in the pathway. Using 69% of all available building blocks, 2597 length-four pathways are assembled, starting from 245 substrates. These substrates are not restricted to external metabolites, since our pathways are not necessarily routes from uptake to secretion.

## Discussion

We conducted five queries using different settings for the parameters as described in section *Scoring function*, corresponding to five different interests. Table 1 summarizes the parameters used.

**Table 1:** The parameter settings and biological emphases in the five queries

	*ω*_*c*_	*ω*_*e*_	*ω*_*f*_	*ω*_*q*_	*Z*_0_	Emphasis
Query 1	0.5	0.5	0.5	0.5	0 for all	overall
Query 2	0	1	0.5	0.5	0 for all	enzyme
Query 3	1	0	0	0	0 for all	compound
Query 4	0.5	0.5	0.5	0.5	100 for "*dg*"and "*eg*", 0 otherwise	gap
Query 5	0	1	1	0	0 for all	enzyme function

In each query, the similarity scores of all 2597 length-four pathways found are computed using (1) and the highest-scoring pathway(s) of a certain substrate is referred as the *best pathway *for that substrate.

It is useful to investigate the building block types as they reflect the differences between species in terms of reactions use, which is not reflected in the scores. Therefore, we categorize the pathways w.r.t. their configurations of building blocks, in order to gain insight in the impact of the parameter settings on the resulting pathway properties. Abbreviations are used to denote the six categories: "*i-i-i-i*" indicates a pathway consists of four "identical" building blocks; "*d*" indicates that the pathway has *at least one *"direct" building block; "*em*", "*dg*", "*eg*" and "*ec*" are defined likewise.

**Figure 4:**
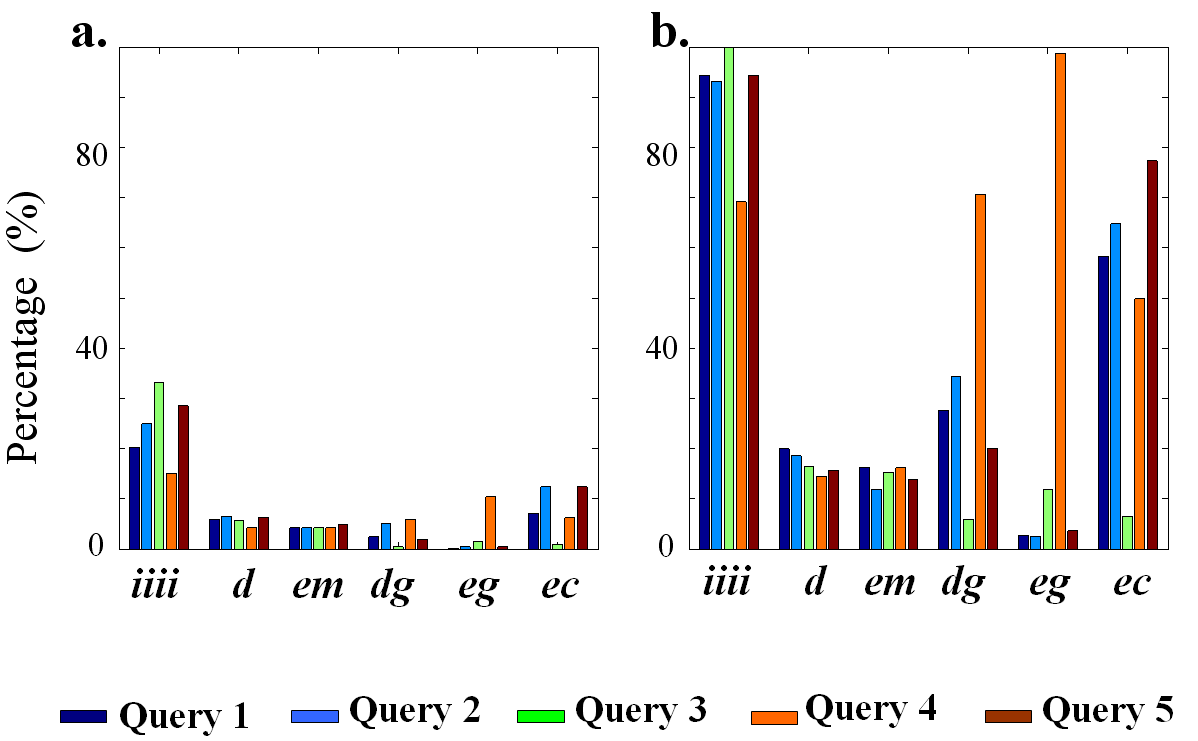
**The percentages of found best pathways in the five queries, with particular pathway configurations**. **a**. Percentage in all pathways with this configuration. **b**. Percentage in all possible best pathways with this configuration. See text for details.

Of all 2597 length-four pathways, 1198 have "*i-i-i-i*" configuration, and 1399 differ between the species, starting from 160 substrates. Among these 426 contain "*d*", 192 "*em*", 199 "*dg*", 709 "*eg*" and 194 "*ec*". For each type of configuration, Figure 4a gives the percentage of best pathways found in all pathways with a particular configuration. Figure 4b corrects the percentages shown in Figure 4a by comparing the number of best pathways with the baseline number of best pathways, which is the maximum possible number of best pathways with that configuration. Therefore Figure 4b actually presents the extent to which a query succeeds in finding a certain type of pathway when only best pathways are concerned.

### The scoring function can address different biological questions

Using our scoring function, different parameter settings result in different best pathways, highlighting different aspects of the pathway features.

Table 1 and Figure 4 can be used as a guide to design a query for a specific purpose. For example, Query 1 finds generally similar pathways in two species. Query 2 only considers enzyme similarity, therefore more best pathways containing "*dg*" and "*ec*" are found (Figure 4). Query 5 is a special case of Query 2, looking for conserved pathways with similar enzyme functions. Compound and enzyme sequence similarities are neglected, thus providing more possibilities for predicting the functions of unannotated genes.

**Figure 5:**
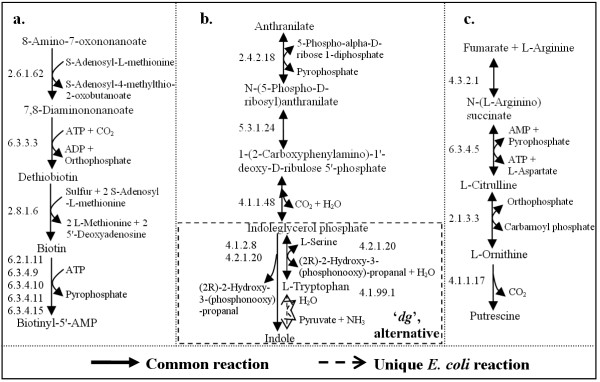
**Examples of the highest-scoring pathways**. **a**. One of the highest-scoring pathways in Query 3, which is involved in biotin metabolism. **b**. One of the highest-scoring pathways in Query 2, but not in Query 1 or Query 4. The last building block is a "*dg*", which contains one unique reaction in *E. coli*, and constitutes an alternative pathway (see text). Involved KEGG maps include: phenylalanine, tyrosine and tryptophan biosynthesis; benzoxazinone biosynthesis; tryptophan metabolism; nitrogen metabolism. **c**. One of the highest-scoring pathways in Query 5, but not in Query 2. Involved KEGG maps include: urea cycle and metabolism of amino groups; alanine and aspartate metabolism; arginine and proline metabolism.

Query 3, on the other hand, considers compound similarity only. If two reactions have the same compounds, they are identical reactions. So all best pathways with "*i-i-i-i*" configuration are found in Query 3 (Figure 4). Identical reactions are highly conserved in the metabolism of different species, and can be used as a measure of phylogenetic distance. Furthermore, those very specific processes containing the most unique compounds will score the highest (see equation 2). Figure 5a shows an example, in which the non-currency compounds are only present in the shown pathway, which is specific to biotin metabolism.

Gaps are preferred in Query 4. Indeed, we can see a large increase in best pathways with "*dg*" and "*eg*" in Figure 4. Moreover, in-depth analysis shows that the numbers of "*dg*" and "*eg*" building blocks in the pathways have also increased four to seven times, demonstrating that the increase of found best pathways with "*dg*" and "*eg*" is not because a limited number of building blocks are used repeatedly. The results may hint at additional intriguing evolutionary phenomena: if one enzyme in species 1 is comparable to the combined functionality of two enzymes in species 2, it may be caused by gene fusion in species 1, or gene duplication in species 2 [13].

### Comparing results of different queries can help infer additional details

It can be instructive to investigate the differences in the results between various queries. For instance, the best pathways of a certain substrate in Query 2 and not found in the best pathways of the same substrate in Query 1 have similar enzymes but use different cofactors or less specific substrates. They are well-conserved, a-specific enzymes. Many pathways containing "*dg*" are found in Query 2 for this reason, as we can see from Figure 4. Figure 5b shows an example, which is found in Query 2 due to its high enzyme similarity, but not in Query 1 or Query 4 for the same substrate due to its low compound similarity. In another example (not shown), a best pathway in Query 2 producing pyruvate is filtered out in Query 1 because pyruvate is less specific, as it is present in 147 reactions [18].

**Figure 6:**
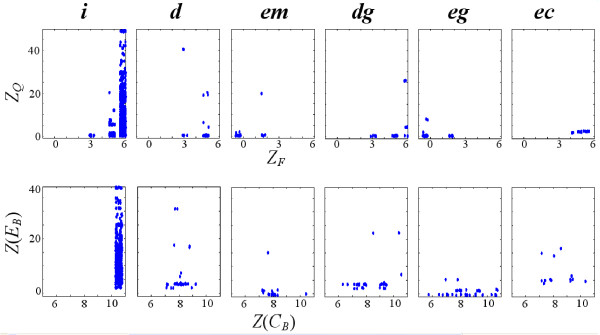
**The distributions of the four component scores for each type of building block**. *Z*_*F *_and *Z*_*Q *_are computed as in (8) and (13). *Z*(*C*_*B*_), *Z*(*E*_*B*_) are computed as in (2) and (7) with the parameter settings of Query 1 (see Table 1).

In addition, the best pathways of a certain substrate in Query 5 and not found in the best pathways of the same substrate in Query 2 have similar enzyme functions but dissimilar enzyme sequences. These enzymes might be non-homologous but evolved into the same function, or the functions have been maintained although their sequences have been changed. An example is given in Figure 5c. The enzymes in the fourth building block, spe1 from *S. cerevisiae *and speC, speF from *E. coli*, have very dissimilar sequences (*E*-value > 100). Although spe1, speC and speF are non-homologous, lysA (EC: 4.1.1.20) in *E. coli *has a sequence similar to that of spe1 (*E*-value = 2.5 × 10^-7^). According to Sandmeier *et al*. [26], speC and speF belong to group III decarboxylases, and spe1 and lysA belong to group IV decarboxylases. Although the homology among the enzymes within each group is established, no evidence has been obtained that the sequences of these two groups are related. Therefore, they seem to have different evolutionary origin. This result demonstrates that enzyme function and sequence do not always correlate with each other. In addition, more "*ec*" are found in Query 5 (see Figure 4) exactly because on average "*ec*" has high enzyme functional similarity but low sequence similarity, as shown in Figure 6.

### Combining the component scores makes sense

Figure 6 presents the component scores of each type of building block and shows that the various information sources are not correlated (see also [9]), making it worthwhile to combine them. In addition, Figure 6 reveals the diverse similarity fingerprint of each type of building block, which calls for further research. For example, the variance of the sequence similarity score in "*i*" is large, which might arise because of different specificity, horizontal gene transfer, gene fusions, or the fact that only subunits of the enzymes are the same. As to "*ec*", their sequences are very dissimilar in spite of their similar functions. Possible reasons could be that the enzymes have different substrate specificities, or that intermediate substrates are very different. They could also have been isoenzymes in parallel pathways, having become specialized to one species during evolution.

### The conserved part of two aligned networks is scale-free

**Figure 7:**
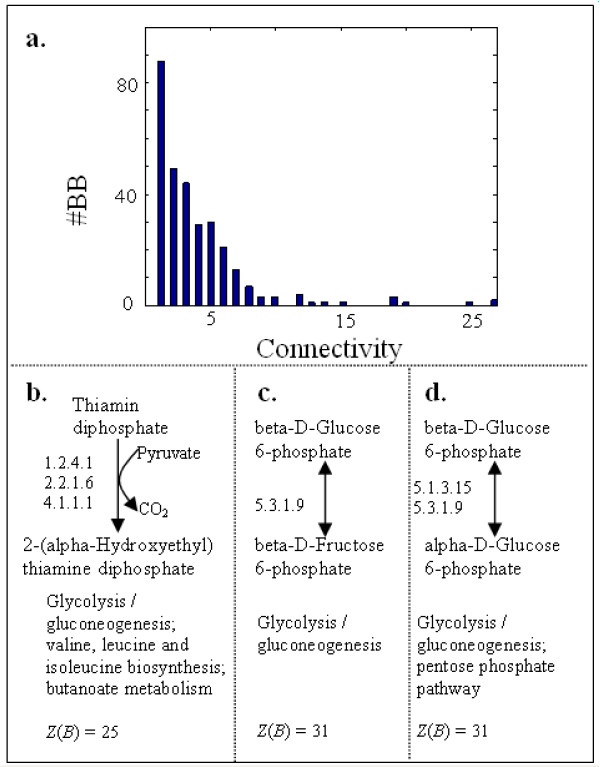
**The building block connectivity**. **a**. Histogram of the number of best pathways in which a building block is involved in Query 1. **b**. – **d**. Three building blocks which are involved in 27, 27 and 25 best pathways in Query 1, respectively. Scores and involved KEGG maps are given underneath the building blocks.

We inspected the connectivity of each building block in Query 1, i.e. the number of best pathways in which a building block is involved. Figure 7a shows that building block connectivity follows a power-law distribution. It has already been pointed out that metabolic networks as a whole are scale-free networks [2]; but our finding provides evidence from a new perspective, indicating that the conserved part of aligned networks, composed of the building blocks in the best pathways, is also scale-free. Figures 7b–d shows the three building blocks with the highest connectivity to be involved in primary metabolism glycolysis/gluconeogenesis, which is known to be highly conserved and plays a role in many processes.

### Short pathways lead to interpretable results

Our methodology has no inherent limit on the pathway lengths. That is, it can construct and score pathways consisting of any number of building blocks. To find longer pathways, one can simply extend the pathway length in the search step. Actually, we conducted experiments without a length limit, which resulted in aligned pathways up to a length of 42 building blocks. Another solution would be to assemble the current length-four short pathways into longer pathways.

However, not all pathway lengths give meaningful results. When the length becomes too short, the method starts to compare individual reactions and loses the power of metabolic functional context, as stated in the background. As a result, some isolated reactions are also included in the results. For example, 31% of building blocks (i.e. length-one pathways) contain isolated reactions, which are not included in any length-four pathway.

**Figure 8:**
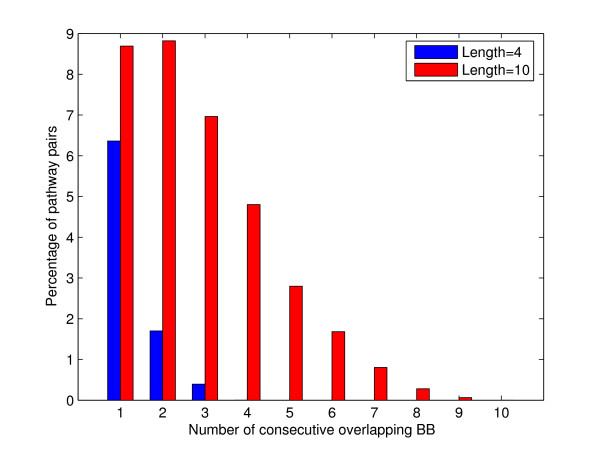
**The impact of pathway length on the resulting overlap**. A frequency graph of the number of consecutive overlapping building blocks in all pairs of pathways of the same length found in Query 1. When pathway length is increased, the overlap between resulting pathways increases significantly, hampering interpretation.

When the pathway length becomes too large, the method produces many highly overlapping results. For example, when running M-PAS with the pathway length set to ten, the number of found pathways increases to 15939 (as compared to the 2597 found pathways when this length is set to four). However, Figure 8 shows that the average overlap between any two pathways also increases significantly. This makes it more difficult to interpret the results. Moreover, longer pathway lengths stress pathway conservation more, and will inevitably miss some interesting short pathways. For example, 128 building blocks (20%) which are present in the results of length-four are not found in the set of length-ten pathways. Therefore, although limiting the pathway length to four might not be the optimal choice, it is within a reasonable range which produces meaningful results.

### M-PAS reveals pathway diversity and alternatives

**Figure 9:**
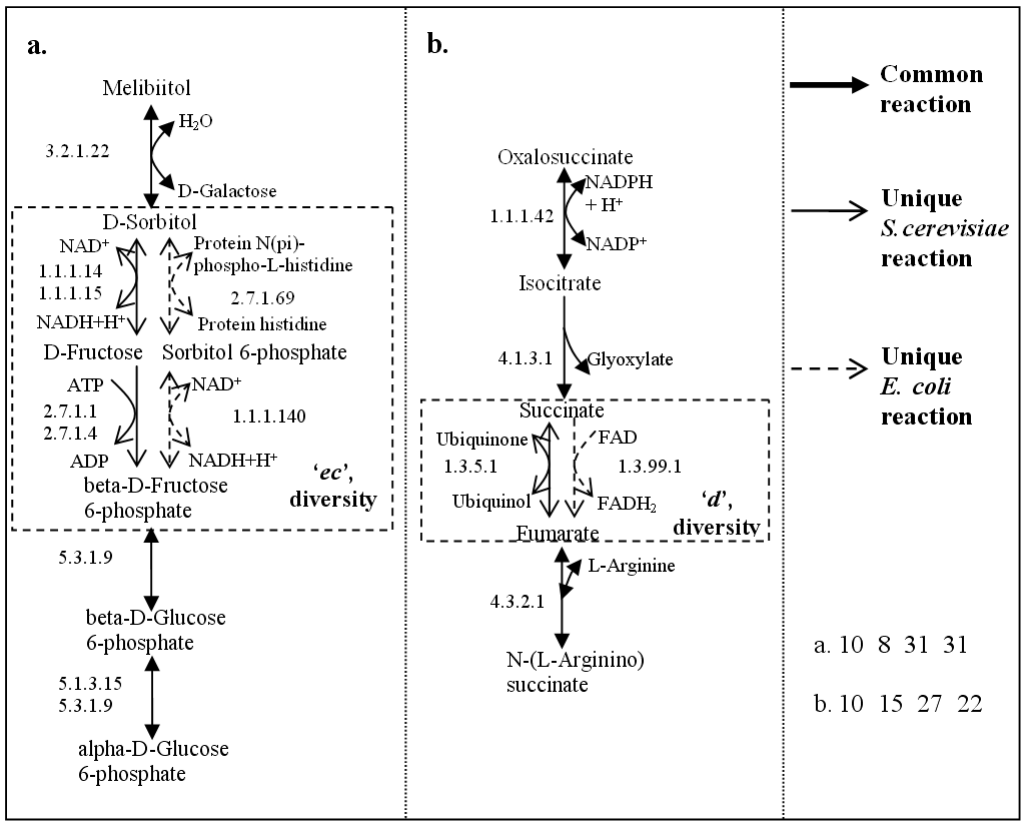
**Two examples of non-"i-i-i-i" best pathways in Query 1**. The non-identical building blocks are highlighted, which exhibit diversities. Scores of all building blocks are shown at the bottom right. The involved KEGG maps are: **a**. galactose metabolism; fructose and mannose metabolism; glycolysis/gluconeogenesis; pentose phosphate pathway. **b**. citrate cycle (TCA cycle); glyoxylate and dicarboxylate metabolism; urea cycle and metabolism of amino groups; alanine and aspartate metabolism; arginine and proline metabolism; butanoate metabolism (only for *E. coli*); reductive carboxylate cycle (CO_2 _fixation) (only for *E. coli*).

As mentioned above, we found that 54% of the length-four pathways are not "*i-i-i-i*", which occur in 65% of the substrates. Interestingly, 17 start substrates do not have any "*i-i-i-i*" pathways, which means the length-four pathways starting with these substrates always differ in these two species. When only best pathways are concerned, we found 16% of these are not "*i-i-i-i*", starting from 13% of the substrates. Figure 9 displays two best pathways in Query 1, which contain unique reactions in both species.

**Figure 10:**
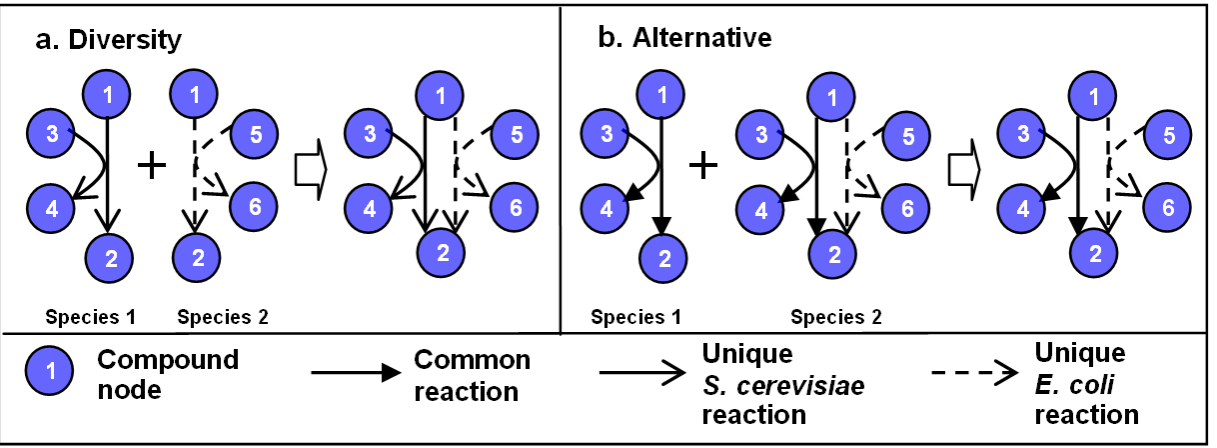
**Illustration of diversity and alternative pathway**. In each case, the reactions in both species are combined into a unified representation for conciseness.

These pathways are highly conserved, yet exhibit differences between the two species. Note that M-PAS goes beyond simple reaction comparison and always places these differences in metabolic functional context. In this way, our method sheds light on variations between species in the use of non-identical but similar reactions in pathways, revealing between-species diversity and within-species alternatives. When both species have their own unique reactions to transform a particular substrate into a particular product, we call this *diversity*. If only one of the species has a unique reaction, which performs the same transformation as another common reaction does in both species, then this unique transformation forms part of an *alternative *pathway. Figure 10 gives a schematic explanation of these two terms, in which different types of arrows are used to indicate unique reactions of one species.

**Figure 11:**
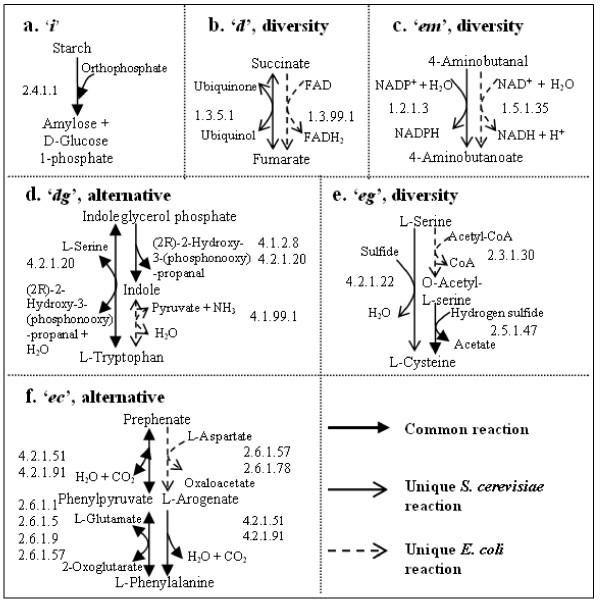
**High-scoring building blocks in Query 1**. **a**. One of the highest-scoring "identical" building blocks (*Z*(*B*) = 34). **b**. One of the highest-scoring "direct" building blocks (*Z*(*B*) = 27). **c**. The highest-scoring "enzyme mismatch" building block (*Z*(*B*) = 16). **d**. The highest-scoring "direct-gap" building block (*Z*(*B*) = 23). **e**. The highest-scoring "enzyme mismatch-gap" building block (*Z*(*B*) = 9). **f**. The highest-scoring "enzyme crossover match" building block (*Z*(*B*) = 18).

Recall the constraint in section *Reaction alignment *which enforces uniqueness in constructing a non-identical building block. Consequently, these non-identical building blocks contain unique reactions in either one or both species, introducing diversity or alternatives in the assembled pathways. In other words, all resulting pathways which do not have an "*i-i-i-i*" configuration contain diversity or alternatives. For example, the fourth building block in Figure 5b contains a reaction unique to *E. coli*, constituting a unique alternative pathway. On the other hand, the second building block in Figure 9a and the third building block in Figure 9b contain unique reactions in both species, therefore they show diversity in the pathways. More examples are given in Figure 11, which displays the most similar building blocks of each type in Query 1.

These results demonstrate the value of including non-identical building blocks, as otherwise these strongly conserved pathways would have been overlooked. In particular, building blocks with gaps or crossovers would be hard to detect manually, e.g. Figure 9a and Figures 11d–f. Take Figure 11d as an example. By comparing reactions in two species, normally we can only find a reversible reaction present in both species which catalyzes indoleglycerol phosphate into L-tryptophan. However, considering gaps allows us to find two consecutive reactions in one of the species which perform the same transformation in two steps. In the end, our algorithm found a unique alternative pathway in *E. col*i which transforms indoleglycerol phosphate to indole first by an irreversible reaction, followed by a unique reaction transforming indole to L-tryptophan.

### New links between different parts of metabolism are found

Our method is global, starting from constructing building blocks to the assembly of pathways. Therefore, the resulting pathways have a reasonable coverage of the network, and explicitly include links between different parts of metabolism, which are displayed in 202 pathway maps of metabolism in KEGG [22]. For example, Figure 9 shows four to seven such maps are linked together in each aligned pathway (see caption).

**Figure 12:**
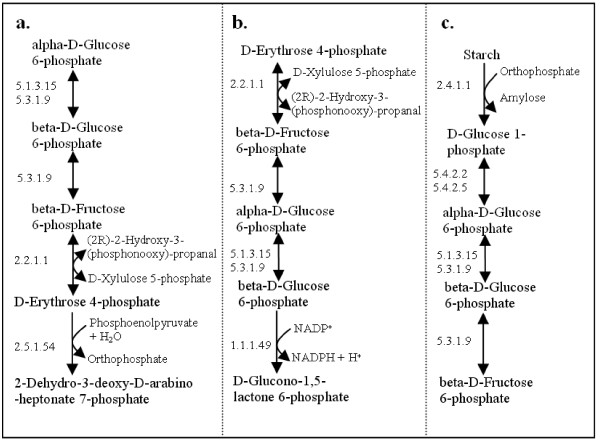
**Three pathways with the highest scores in Query 1**. For conciseness, a common reaction in *S. cerevisiae *and *E. coli *is drawn only once in each building block. The solid-headed arrow indicates the reactions exist in both species, constituting an "*i-i-i-i*" pathway. **a**. *Z*(*P*) = 41. Involved KEGG maps include: glycolysis/gluconeogenesis; pentose phosphate pathway; starch and sucrose metabolism; phenylalanine, tyrosine and tryptophan biosynthesis. **b**. *Z*(*P*) = 40. Involved KEGG maps include: pentose phosphate pathway; glycolysis/gluconeogenesis; starch and sucrose metabolism; glutathione metabolism. **c**. *Z*(*P*) = 39. Involved KEGG maps include: starch and sucrose metabolism; glycolysis/gluconeogenesis; galactose metabolism; streptomycin biosynthesis; pentose phosphate pathway.

Since our alignment method operates on individual reactions, independent of the existing pathways as given in current databases, we not only reconstruct known pathways (as presented by KEGG, e.g. Figure 5, 9a, 12b–c), but also discover new pathway possibilities with the component reactions annotated in different maps and not linked with each other in the original database, e.g. Figure 9b and 12a. These pathways will not be found if we only look at the pathways shown in the maps and the links between maps.

Moreover, M-PAS not only links different parts of metabolism within one species, but also associates diverse parts in two species with each other, offering potential interesting targets for bioengineering. For instance, in Figure 11e, the unique reaction of *S. cerevisiae *is found in glycine, serine and threonine metabolism, while the unique reaction of *E. coli *is found in cysteine metabolism. Therefore it will not be found if we only look at one map or one species at a time.

### Primary metabolism is highly conserved

Three pathways with the highest scores in Query 1 are shown in Figure 12. They represent the most conserved part of the metabolic network in the two species and are therefore expected to be important. Not surprisingly, the three pathways are all involved in primary metabolism. Moreover, they all have "*i-i-i-i*" configuration, meaning all reactions in the pathways are conserved across species. Clement *et al*. [27] also pointed out that "vital biological processes in a group of related species should be conserved and expressed by a significant number of reactions in all the organisms of the group".

We can also observe this in Figure 11, where the involved parts of metabolism in the highest-scoring building blocks are rather central processes, e.g. starch and sucrose metabolism, citrate cycle (TCA cycle), CO_2 _fixation and other important amino acid metabolisms.

## Conclusion

In this work, we extend our former alignment framework and propose a novel scoring method to identify conserved metabolic pathways and quantify the level of conservation in an efficient and comprehensive manner. Based on the six types of building blocks, a systematic search is conducted in the network. We find and rank conserved pathways given certain substrates, and shed light on the variations between species within a metabolic functional context. This is not possible by simple comparison of reaction lists or enzyme lists.

Our method combines individual reactions, so that we can find conserved pathways that are not represented in conventional databases. Since the alignment and search are conducted in the whole network, M-PAS unites reactions in different KEGG maps, revealing links and relating reactions with common upstream substrates and downstream products which might be elusive if we only look at subsets of the network.

Our similarity measure combines uncorrelated information sources, including similarities of substrate sets, product sets, enzyme functions, enzyme sequences and alignment configurations. The function has a generic form and is capable of measuring pathway similarity given different biological emphases. Due to its hierarchical integration structure, it is readily extensible to include other relevant similarity measures if available (e.g. enzyme affinities), or to modify a component score (e.g. using compound molecular similarity scores). Moreover, the proposed function is plausible since parts of primary metabolism, which are known to be well conserved, are found to be abundant in our top-scoring pathways and building blocks.

M-PAS reveals highly conserved pathways containing diversity or alternatives, which yields important information for biology and life sciences. First, the results give insight into the evolutionary differences between species. For instance, the two species apparently diverged to process 17 substrates differently, so that no "*i-i-i-i*" pathways are found starting from them. This divergence calls for special treatment of these substrates per species in analysis and applications. Second, the diversity and alternatives in conserved pathways also provide additional ways to construct metabolic networks for currently unannotated species. Third, our analysis lists potential candidate enzymes for bioengineering, i.e. certain natural enzymes can be removed, introduced, or changed so that we can select a favorable pathway to enforce production of a metabolite of interest, or block pathways leading to certain unfavorable products. In particular, alternative pathways have to be considered in drug design, because blocking central enzymes might not be effective when alternative pathways provide other routes, and cause drug resistance in the pathogen population [1].

M-PAS is currently constrained to finding linear pathways which are strictly similar. Although further processing these linear pathways, e.g. combining them, could reconstruct some tree-like subnets and cycles, not all network structures can be captured. M-PAS could be extended to construct and score more complex pathway topologies that capture more variation. First, to capture more variation, one may extend the building block definition to include larger differences, e.g. a '*dg*' with two gaps, or to allow compound mismatch. But care needs to be taken to keep the computational load acceptable and to avoid linking unrelated pathways. Alternatively, one may reduce the pathway length, e.g. to assemble two building blocks into a pathway to capture diverse pathways with short overlaps. However, as discussed earlier, when the pathway length becomes too short, the method starts to compare individual reactions. To find more complex pathway topologies, a more complex search algorithm is required. An alternative would be to expand our building block definition to incorporate more types of network motifs. But again, the computational load will increase significantly.

The complementary reaction information of multiple well-studied model species provides more confidence and more possibilities to transfer this information to a new species. Although M-PAS currently only performs pairwise alignment on two species, we expect even more informative results when it is applied on multiple species, and larger differences will be found as the phylogenetic distance increases. Finally, by relating different sets of enzymes in different species to a common metabolic function, this work provides an infrastructure in which regulatory factors can be incorporated, and functional hypotheses can be generated.

## Authors' contributions

YL developed and implemented the method, performed the analysis and wrote the manuscript. DR and MR supervised the work, contributed substantially in its design and revised the manuscript. MG helped to analyse results and to revise the manuscript. MR conceived the research. All authors read and approved the final manuscript.

## Appendix

The twenty-six currency metabolites are ATP, ADP, UTP, UDP, GTP, GDP, AMP, UMP, GMP, NAD, NADH, NADP, NADPH, acetyl-CoA, CoA, propanoyl-CoA, L-glutamine, L-glutamate, 2-oxoglutarate, CTP, CDP, CMP, H_2_O, CO_2_, NH_2_, and phosphate.
